# Poncet’s disease after the intravesical instillation of Bacillus Calmette–Guérin (BCG): a case report

**DOI:** 10.1186/s13104-017-2606-9

**Published:** 2017-08-18

**Authors:** Paula Cíntia Machado Sampaio, Yan Garcia Lira, Hellen Yuki Umemura Ribeiro, Fernanda de Paula Moreira, Maitê Silva Martins Gadelha, Sérgio Ferreira Santos da Cruz

**Affiliations:** 1 Section of Oncological Treatment Research, Division of Oncology, Department of Clinical Care, Centro de Tratamento Oncológico, 4402 Mundurucus Street, Guamá, Belém, Pará 66063-495 Brazil; 2 Section of Chemotherapy, Division of Oncology, Department of Clinical Care, Hospital Ophir Loyola, 992 Magalhães Barata Avenue, São Braz, Belém, Pará 66000-000 Brazil; 3 Section of Clinical Oncology, Division of Oncology, Department of Clinical Care, Universidade do Estado do Pará, 2623 Perebebuí Street, Marco, Belém, Pará 66087-670 Brazil; 4 Section of Oncology, Division of Clinical Care, Department of Evidence-Based Medicine, Universidade do Estado do Pará, 2623 Perebebuí Street, Marco, Belém, Pará 66087-670 Brazil; 5 Section of Clinical Oncology, Division of Oncology, Department of Clinical Care, Centro Universitário do Pará, 3775 Almirante Barroso Avenue, Souza, Belém, Pará 66613-903 Brazil; 6 400 Senador Lemos Avenue, Campina, Belém, Pará 66050-000 Brazil

**Keywords:** Poncet’s disease, Reactive arthritis, Urothelial carcinoma, Bladder cancer, Intravesical Calmette–Guérin Bacillus, Case report

## Abstract

**Background:**

Poncet’s disease is a rare syndrome characterized by articular impairment in a form of rare tuberculid. One of the theories of its cause involves an autoimmune response induced by the intravesical administration of the Calmette–Guerin Bacillus or the treatment of bladder carcinoma. Furthermore, there may be an appearance of oligoarticular or polyarticular arthritis, beginning 1–3 months after the start of therapy. Few physicians know the disease and the literature related to that syndrome is scarce and restricted to case reports, which contributes to its under diagnosis.

**Case presentation:**

Female patient, 64 years old, Caucasian, in whom was noticed firstly dark urine, without haematuria or dysuria. Later felt also colic pain in the hypogastric region. Microscopically, the conclusive diagnosis was a high grade non-invasive papillary urothelial carcinoma. Thereupon, the treatment of the tumour began with transurethral resection technique and intravesical instillation of Calmette–Guérin Bacillus as adjuvant treatment. Eight months after the beginning of treatment, the lingering presence of the carcinoma was identified. Nevertheless, arthritis was identified through radiographs, after an increase in the clavicle capitation, right knee and left ankle in bone scintigraphy. Coinciding with the joint manifestations, the patient developed fever and purulent urethral discharge (culture was negative). Therefore, trying to investigate the cause of the arthritis, Purified Protein Derivate was taken, with reactive results. An increase of acute phase reactants was found, with other tests resulting normal: blood chemistry, Complete Blood Count, immunology and serology. Human Leukocyte Antigen typing by polymerase chain reaction revealed the presence of A24/AX, B44, B27, BW4/BW4, DQ7 and DQ5. Consequently, Poncet’s disease was the diagnostic conclusion. The treatment with intravesical Calmette–Guérin Bacillus was immediately discontinued. The patient received corticosteroids associated with etoricoxib and isoniazid for 4 months, achieving disappearance of the inflammatory joint signs in 3 months. After 6 months, no joint pain recurrence or other manifestations suggesting active disease had been seen.

**Conclusions:**

Therefore, such diagnosis should be considered when confronted with an osteoarticular clinical picture in patients treated with intravesical Calmette–Guérin Bacillus, especially patients with HLA-B27 (+) and B7 (+), as Poncet’s disease is a reactive arthritis.

## Background

Tuberculous rheumatism, also known as Poncet’s disease, is a rare syndrome described in 1897 by the Frenchman Poncet [1]. It is characterized by articular impairment in a form of rare tuberculid, not related to direct invasion by the micro-organism, but to an immune reaction to the tuberculo-protein (tuberculin hiperergia), constituting a reactive arthritis (ReA) [2–4]. Understanding the pathogenesis of tuberculous rheumatism is still a little explored topic, rightly controversial [2].

Molecular mimicry and proteins heat shock are theories that can be employed to try to understand the pathogenesis of Poncet’s disease [2, 4–7]. In the molecular mimicry, there is an interaction between antigens of the infectious agent, which is mycobacteria in this case, and components of joint tissue. One of the theories involves an autoimmune response induced by the intravesical administration of the Calmette–Guerin Bacillus (BCG) for the treatment of bladder carcinoma that occurs more frequently in patients with Human Leukocyte Antigen HLA-B27 (+) (nearly 60% of reported cases) or B7 (+) (which shows strong affinity with HLA-B27) [1].

Furthermore, there may be, in these cases, an appearance of oligoarticular or polyarticular arthritis in 3% of treated individuals, 1–3 months after the start of therapy [6]. The arthropathy is externalized as oligoarticular pain, mono or polyarticular, acute or subacute character and of variable intensity, uneven distribution, with no signs of redness, heat, without references to pain and morning stiffness, having possibly algid limitation. It reaches hands, wrists, knees, feet, ankles, shoulders, elbows and hips, in descending order, and, exceptionally, the temporomandibular joints [8]. The differential diagnosis is made with chronic rheumatisms, especially rheumatoid arthritis, collagenases, infectious arthritis and arthritis by Koch’s bacillus [9].

For treatment, desensitization to tuberculin, tuberculostatic drugs, nonsteroidal anti-inflammatory drugs (NSAIDs) and the antibiotic scheme for tuberculosis are used [2, 10–12]. The collateral effects are divided in two groups: lower and greater intensity. The lower intensity encompasses nausea, burning sensation, numbness in the feet and hands, drowsiness and colour changes in the urine. The greater intensity includes skin rash with or without pruritus, vertigo, nystagmus, hepatitis, visual changes, shock, purpura, acute renal failure and oliguria [13, 14].

Few physicians know the disease and the literature related to that syndrome is scarce and restricted to case reports, which contributes to its under diagnosis.

## Case presentation

Female patient, 64 years old, Caucasian, with no history of diabetes, tuberculosis, smoking or alcohol abuse, leprosy, lives in the city of Belém, capital of Pará, Brazil. Noticed firstly was dark urine, without haematuria or dysuria. Later, the patient also felt colic pain in the hypogastric region, which led to her admission at the hospital in January 2015, where exams were taken, such as the bladder anatomopathological exam, using the transurethral resection technique (TUR) for analysis of a possible tumour. The existence of the tumour was confirmed and, initially, it had as macroscopic characteristics: irregular tissue fragments, mousy, brownish areas, firm and elastic permeating clogs, weighing combined 5 g and measuring 4.5 × 1.0 cm; microscopically, the tumour showed papillary fragments made of vascular-connective axis, sometimes slender and sometimes wide, lined with several layers of urothelial cells with oval or slightly irregular nuclei, a little augmented in volume and hipercoloured. The diagnosis consisted of a low grade papillary urothelial carcinoma, without clear evidence of invasion and with absence of elements from the proper muscular layer in the examined cuts.

Roughly a month and a half after the diagnosis, in March 2015, a new anatomopathological was realized with the TUR under bladder anaesthesia, which macroscopically showed various irregular fragments of tissue of brownish colour and fibro-elastic consistency, which combined weighed 27 g and overlaid and area of 9.0 × 6.5 × 2.0 cm. Microscopically, there was an integration between the macroscopic and the conclusive diagnosis, which was a non-invasive papillary urothelial carcinoma, but high grade.

That way, the treatment of the tumour began with the transurethral resection technique (TUR) and intravesical BCG as adjuvant treatment. At the month of the conclusive diagnosis, it was started treatment with 40 mg of intravesical BCG, once a week for 6 weeks, as an attack dose. Afterwards, began the second phase: every 3 months during the first semester of treatment, with medication administered once a week, for three consecutive weeks. Finally, the treatment was supposed to be applied every 6 months until 24 months after the TUR.

However, 8 months after the beginning of treatment, accompaniment exams where taken, such as radiograph and anatomopathological, that identified the lingering presence of the non-invasive high grade papillary urothelial bladder carcinoma. Other exams included: an ultrasound of the urinary tract, which pointed to solid nodules on the posterior and lateral walls; and a complete abdomen Computed Tomography (CT), that pointed to pleural effusion on the left base and kidney angiomyolipoma of up to one centimetre. Furthermore, Complete Blood Count (CBC), creatinine, sodium and potassium were within regular levels.

**Fig. 1:**
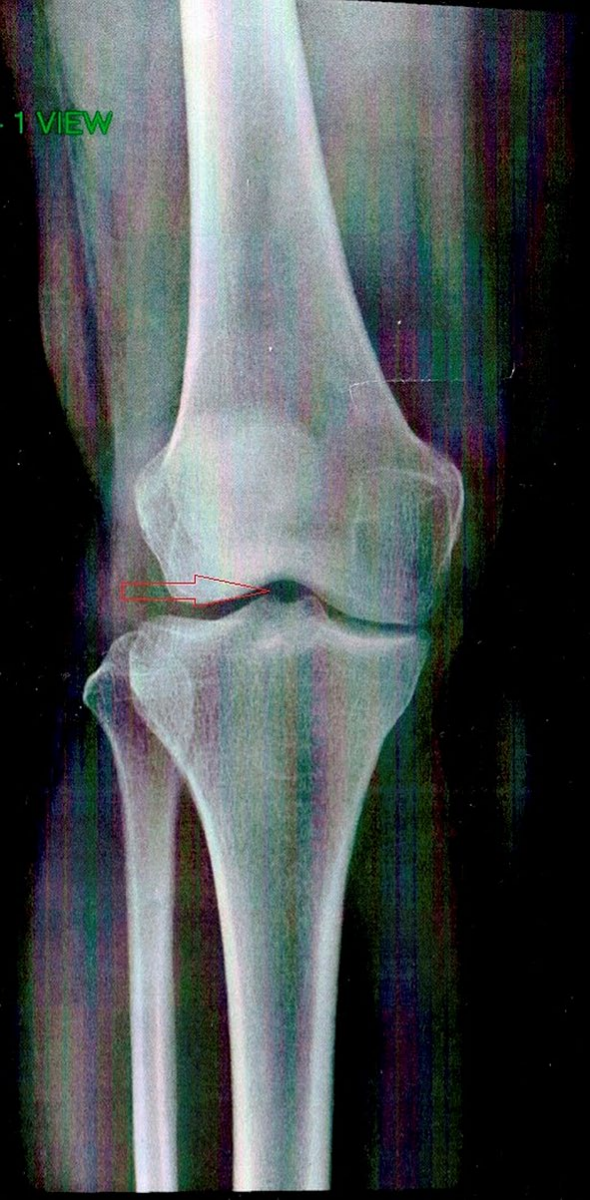
Right knee radiograph: medial compartment tibiofemoral joint space narrowing. Patient presented with dactylitis, intense pain in asymmetrical areas (clavicle, right knee and left ankle) with increased capitation in bone scintigraphy. The right knee radiograph displayed medial compartment tibiofemoral joint narrowing in the right knee, indicated by the *arrow in the figure*, without pathological calcifications or radiographic bone density and texture alterations

**Fig. 2:**
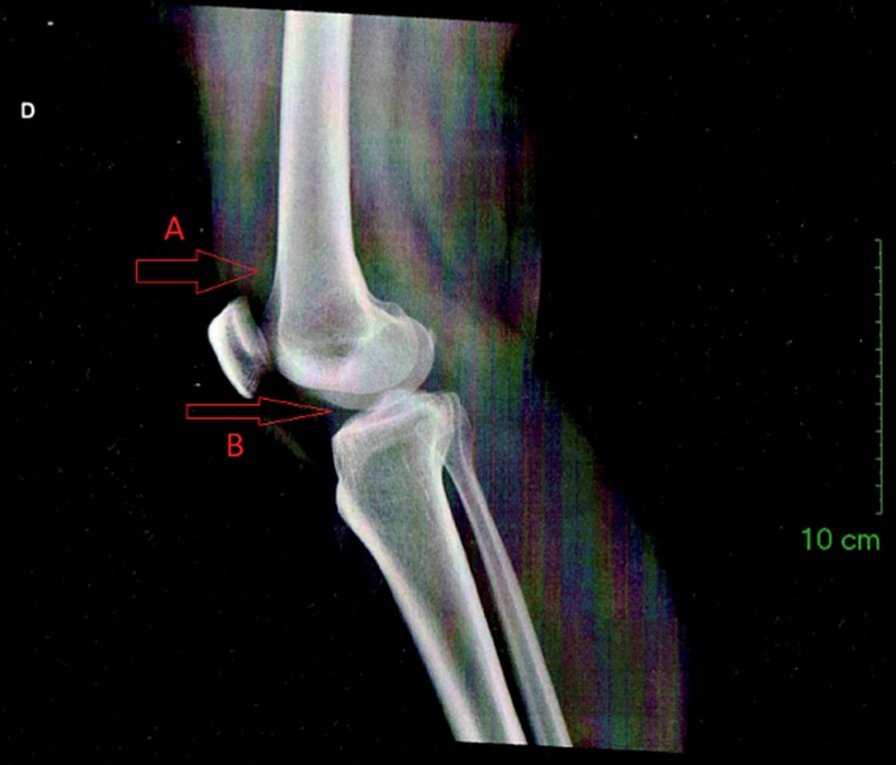
Right knee radiograph: suprapatellar synovial bursa opacification. The right knee radiograph displayed opacification of the suprapatellar synovial bursa, which is indicated by the *arrow A in the figure*, along with the narrowing of the medial compartment tibiofemoral joint in the right knee, indicated by the *arrow B in the figure*, and clinical picture, without any other alteration, indicates joint effusion, synovitis and active arthritis

**Fig. 3:**
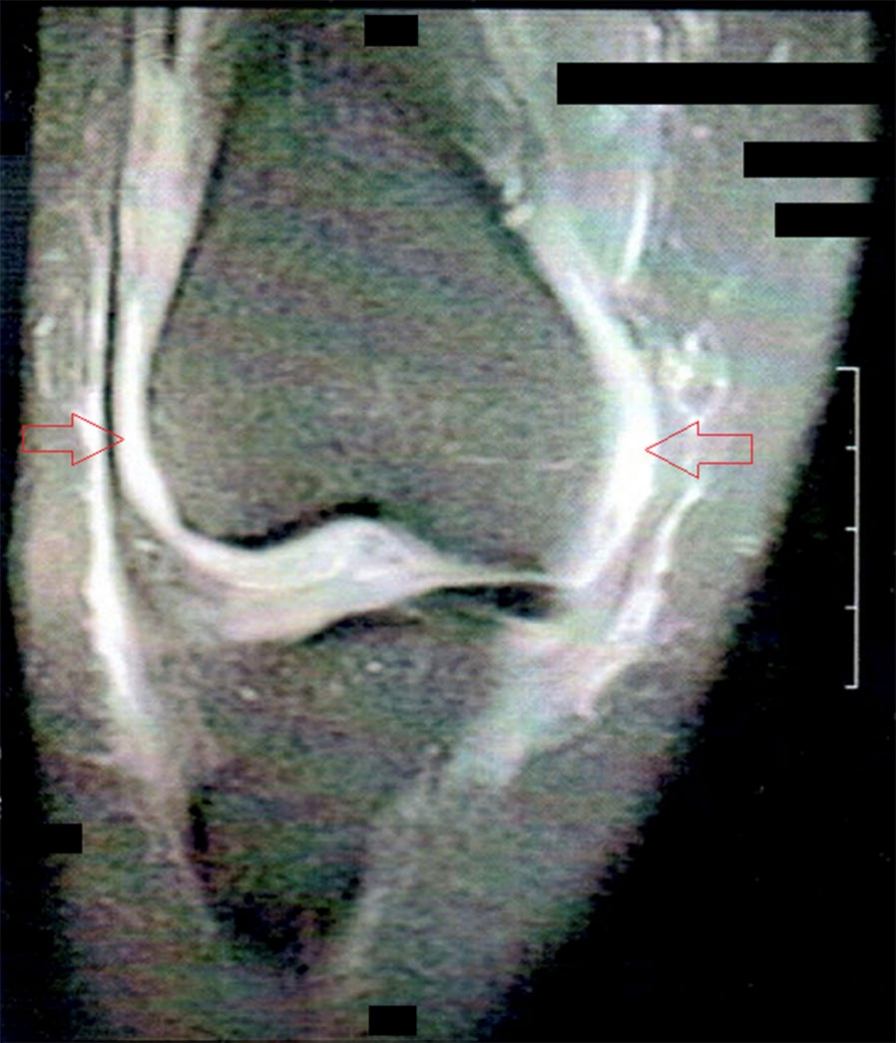
Right knee magnetic resonance image: joint effusion with synovitis signs. Patient presented with intense pain on the right knee, with increased capitation in bone scintigraphy, medial compartment tibiofemoral joint space narrowing and suprapatellar synovial bursa opacification in the radiograph. The magnetic resonance image showed joint effusion and signs of synovitis, indicated by *both arrows in the figure*, without any other alteration, along with the clinical picture, indicate active arthritis

Nevertheless, it was identified through bone scintigraphy an increase in the clavicle capitation, right knee and left ankle. Considering that, active arthritis was later identified by Radiographs (Figs. 1,  2) and Magnetic Resonance Imaging (MRI) of the right knee (Fig. 3). The exams’ results were added to the complaints of dactylitis and intense pain, asymmetrical in the areas mentioned in the exam.

Coinciding with the joint manifestations, the patient developed fever (up 38.2 °C, without associated dysthermia) and purulent urethral discharge. From the analytical point of view, she showed an increase of acute phase reactants (C reactive protein 98.60 mg/l, erythrocyte sedimentation rate 97 mm/l; first hour), with other tests resulting normal, including blood chemistry, CBC, immunology (rheumatoid factor, antinuclear antibody, complement and immunoglobulins) and serology (Chlamydia, Salmonella, human immunodeficiency virus, hepatitis viruses B and C). The culture of the urethral discharge was also negative. HLA typing was performed for HLA-A, B, DR and DQ by PCR (HLA-DRB1 and HLA-DQB1 subtypes were performed by sequence-specific oligonucleotide probe hybridization) and revealed the presence of A24/AX, B44, B27, BW4/BW4, DQ7 and DQ5.

Therefore, it was tried to investigate the cause of the arthritis at the ankle. The Purified Protein Derivative (PPD) was taken, which showed the result of 18 mm (reactive). Because of the temporal relationship between the intravesical BCG instillation and the onset of dactylitis and the asymmetrical oligoarthritis with urethritis, fever and HLA-B27 (+), we established the diagnosis of Poncet’s disease secondary to BCG. The treatment with intravesical instillation of BCG was immediately discontinued. The patient initially received low-dose corticosteroids (prednisone 5 mg/day), without any improvement, so we indicated etoricoxib 90 mg/day and isoniazid 300 mg/day, with treatment lasting up to 4 months, between October 2015 and January 2016, achieving progressive control and complete disappearance of the inflammatory joint signs in 3 months. After 6 months (March 2016), no joint remission (axial or peripheral) or other manifestations suggesting active disease had been seen.

## Discussion

Intravesical instillations of the Calmette–Guerin Bacillus (BCG), as local immunotherapy, has been used since 1976 in patients with intermediate and high grade superficial bladder carcinoma, with its use being widespread at present and having proven safe and effective [15]. Its antitumor activity concentration at the site of instillation does not make this treatment incapable of inducing non-serious systemic side effects and self-limiting fever and pain in up to 5% of patients [15, 16]. Osteoarticular side effects are rare, being described in 1–5%, mainly joint pain and arthritis in 0.5–1% of patients [17, 18]. We report the case of a patient who, after receiving intravesical instillations of BCG for the treatment of bladder carcinoma, developed tuberculous rheumatism, also known as s arthritis, constituting reactive arthritis.

The development of musculoskeletal side effects secondary to intravesical BCG instillation for the treatment of bladder cancer is very rare considering its wide distribution and the number of patients under treatment, reducing its frequency to case reports and small series [3]. Tinazzi et al. conducted a systematic review including cases of autoimmune manifestations related to the intravesical administration of BCG. Results showed joint pain and/or arthritis in 64% of patients, Reiter’s syndrome in 24%, arthritis and fever in 4% and psoriatic arthritis in 2% [15].

Poncet’s arthritis secondary to BCG usually occurs in men between 50 and 60 years of age, manifesting as asymmetric arthritis, normally found in wrists, ankles and knees, and fever associated in more than half of cases [19]. Dactylitis [20] and urethritis [21] have also been described as part of this clinical picture. It develops mostly after the fourth or fifth instillation. Joint fluid has inflammatory characteristics with predominance of polymorphonuclear cells and mycobacteria cultures obtained from the joint fluid, urine and blood are negative (thus excluding the possibility of septic arthritis by BCG, which has also been described), complementary examinations usually shows a moderate increase in acute phase reactants [17, 19].

It has been suggested that the mechanism by which the instillation of BCG induces Poncet’s disease is molecular mimicry, once the heat shock protein HSP65 of mycobacteria shares homology with a human cartilage proteoglycan, and also presents cross-reactivity with the haplotypes of HLA-DR1, DR3 and DR4, stimulating the secretion of cytokines and activation of CD8 (+) [20]. The autoimmune response induced by the administration of BCG occurs more frequently in patients with HLA-B27 (+) (nearly 60% of reported cases) or B7 (+) (which shows strong affinity with HLA-B27) [1].

The co-relation between HLA-B27 (+) and arthritogenic peptides has been vastly demonstrated in the literature [22–25], being considered, even after 30 years of its discovery, one of the best genetic markers to date, regarding a major histocompatibility complex antigen [22]. As to its relevance on patients with Poncet’s disease, a case–control Mexican study presented p = 0.01 when studying 16 patients diagnosed with the aforementioned form of arthritis against 99 healthy individuals [7], therefore proving this genetic susceptibility. This line of action has also been found on case reports, exposing the importance of HLA tests on patients with osteoarticular clinical picture [24, 25].

Most patients with secondary Poncet’s arthritis respond favourably to complete suspension of BCG treatment. The disease may become chronic in a small percentage, requiring specific therapy, such as NSAIDs and corticosteroids used alone or in combination, rarely with association of immunosuppressants, such as methotrexate. In patients with an inadequate response to these treatments, as in our case, some authors suggest the addition of anti-tuberculosis (TB) drug treatment [1, 24, 25] as isoniazid, 5 mg/kg with maximum dose: 300 mg/day [26], which was used by the patient. Although this therapy is controversial, some chronic and refractory cases have been reported solved after the use of isoniazid for 3 months [27, 28].

## Conclusions

Poncet’s disease as a reactive arthritis has been previously described as a possible and rare complication of the immunotherapy with intravesical instillation of BCG for bladder carcinoma, especially in patients with HLA-B27 (+) and B7 (+). Therefore, this diagnosis should be considered when confronted with and osteoarticular clinical picture in patients treated with BCG. Physicians should be aware of the appropriate treatment used for Poncet’s disease and its collateral effects for better patient care.

## Abbreviations

ReA: reactive arthritis
BCG: Calmette–Guerin Bacillus
TUR: transurethral resection
CT: computed tomography
CBC: complete blood count
HLA: human leukocyte antigen
PPD: purified protein derivative
NSAIDs: nonsteroidal anti-inflammatory drugs
TB: tuberculosis

## References

[1] Bloxham CA, Addy DP (1978). Poncet’s disease: para-infective tuberculous polyarthropathy. Br Med J.

[2] Sincock DE, Mukherjee D, Gendi NST (2004). Poncet’s disease—a novel cause of non-compliance with anti-tuberculous drugs. Respir Med.

[3] Maricic MJ, Alepa FP (1990). Reactive arthritis after *Mycobacterium avium* intracellular infection: Poncet’s disease revisited—short communication. Am J Med.

[4] Dall L, Long L, Stanford J (1989). Poncet’s disease: tuberculous rheumatism. Rev Infect Dis.

[5] Issacs AJ, Sturrock RD (1974). Poncet’s disease-fact or fiction?. Tuber Lung Dis.

[6] Tischler M, Schonfeld Y (1996). Tuberculose et autoimmunité: òu en sommes—nous?. Annales de L´Institut Pasteur.

[7] Southwood TR, Gaston JSH (1990). The molecular basis for Poncet’s disease. Br J Rheumatol.

[8] Valleala H, Tuuminen T, Repo H, Eklund K, Leirisalo-Repo M (2009). A case of Poncet disease diagnosed with interferon-γ- release assays. Nat Rev Rheumatol.

[9] Kritski AL, Conde MB, Souza GRM (2005). Tuberculose: do ambulatório à enfermaria.

[10] Mariani B (1969). Present aplications of tuberculin desensitizing therapy. Folia Allergol (Roma)..

[11] Schweitzer LC, Lipnharski F, Prezzi SH (2011). Artrite de Poncet: relato de caso. Rev Bras Reumatol..

[12] Sharma A, Pinto B, Dogra S, Sharma K, Goyal P, Sagar V (2016). A case series and review of Poncet’s disease, and utility of current diagnostic criteria. Int J Rheum Dis..

[13] Arbex MA, Varella MCL, Siqueira HR, Mello FAF (2010). Antituberculosis drugs: drug interactions, adverse effects, and use in special situations. Part 2: second line drugs. J Bras Pneumol..

[14] Pereira JCB (2005). When we shall use tuberculin desensitizing therapy. Pulmão RJ..

[15] Tinazzi E, Ficarra V, Simeoni S, Artisan W, Lunardi C (2006). Reactive arthritis following BCG immunotherapy for urinary bladder carcinoma: a systematic review. Rheumatol Int.

[16] Lamm DL, Van Der Meijden PM, Morales A, Brosman SA, Catalona WJ, Herr HW (1992). Incidence and treatment of complications of bacillus Calmette-Guerin intravesical therapy in superficial bladder cancer. J Urol..

[17] Clavel G, Grados F, Lefauveau P, Fardellone P. Osteoarticular side effects of BCG therapy. Joint Bone Spine. 2006;73(1):24–8. 10.1016/j.jbspin.2004.12.003 16461205

[18] Orihuela E, Herr HW, Pinsky CM, Whitmore WF Jr. Toxicity of intravesical BCG and its management in patients with superficial bladder tumors. Cancer. 1987;60(3):326–33. 10.1002/1097-0142(19870801)60:3<326::aid-cncr2820600309>3.0.co;2-5 3594369

[19] García PH, Mateos CB, Arnés MC. Artritis reactiva secundaria a inmunoterapia intravesical con bacilo de Calmette-Guerin. Semergen. 2006; 32(4):176–8.

[20] Manzini CU, Bernini L, Elkhaldi N, Mascia MT, Ferri C (2006). Dactilitis and oligoarthritis after BCG immunotherapy in a patient affected by bladder cancer. Reumatismo..

[21] Belmatoug N, Levy-Djebbour S, Appelboom T, De Gennes C, Peltier AP, Meyer O (1993). Polyarthritis in 4 patients treated with intravesical BCG-therapy for carcinoma of the bladder. Rev Rhum Ed Fr..

[22] Hameed K, Karim M, Islam N, Gibson T (1993). The diagnosis of Poncet’s disease. Br J Rheumatol.

[23] Pardalidis NP, Papatsoris AG, Kosmaoglou EV, Georganas C (2002). Two cases of acute polyarthritis secondary to intravesical BCG adjuvant therapy for superficial bladder cancer. Clin Rheumatol.

[24] Kroot EJ, Hazes JM, Colin EM, Dolhain RJ (2007). Poncet’s disease: reactive arthritis accompanying tuberculosis. Two case reports and a review of the literature. Rheumatology (Oxford).

[25] Irmi ZI, Zaiton A, Faezah H (2013). Reactive arthritis in tuberculosis: a case of Poncet’s disease. Malays Fam Phys..

[26] Reves R, Heilig CM, Tapy JM, Bozeman L, Kyle RP, Hamilton CD (2014). Intermittent tuberculosis treatment for patients with isoniazid intolerance or drug resistance. Int J Tuberc Lung Dis..

[27] Milas L, Maillefert JF, Michel F, Piroth C, Wautot A, Tavernier C (1999). Refractory arthropathy after intravesical bacillus Calmette–Guérin therapy. Usefulness of isoniazide. Rev Rhum Engl Ed.

[28] Neumayr C, Kirchgatterer A, Knoflach P (2002). Chronic reactive arthritis associated with Calmette–Guérin bacillus. Dtsch Med Wochenschr.

